# Solubility prediction in the bRo5 chemical space: where are we right now?

**DOI:** 10.5599/admet.834

**Published:** 2020-07-08

**Authors:** Giuseppe Ermondi, Vasanthanathan Poongavanam, Maura Vallaro, Jan Kihlberg, Giulia Caron

**Affiliations:** 1 Department of Molecular Biotechnology and Health Sciences, University of Torino, Quarello 15, 10135, Torino, Italy; 2 Department of Chemistry - BMC, Uppsala University, SE-75123, Uppsala, Sweden

**Keywords:** Chameleonicity, flexibility, ionization, lipophilicity, polarity

## Abstract

Modelling the solubility of compounds in the “beyond Rule of 5” (bRo5) chemical space is in its infancy and to date only a few studies have been reported in the literature. Based on our own results, and those already published, we conclude that consideration of conformational flexibility and chameleon like behaviour is important, but quantitative models that account for these properties remain to be developed. Inclusion of 3D information appears to be somewhat less important than for cell permeability and extremely challenging due to the difficulties of accurate conformational sampling in the bRo5 space. Currently, methods for modelling of solubility will have to be tailored to the set of investigated compounds.

## Solubility, and solubility in the bRo5 chemical space

Aqueous solubility plays a crucial role in filtering lead compounds and drug candidates in early stages of drug discovery and development [1]. Although dependent on the research program, GSK on the basis of the General Solubility Equation (GSE, see below) recently suggested that solubility is satisfactory (high) when >200 μM, while 30–200 μM was considered as intermediate and <30 μM as poor [2].

Application of *in silico* methods is one of the most appealing strategies to overcome solubility issues in drug discovery projects [3]. However, predicting solubility is not an easy task mainly because of the high uncertainty affecting the experimental data [4], with typical interlaboratory measurement reproducibilities of 0.6 log *S* units (with *S* in mol/L) [5]. The main approaches for prediction of solubility have recently been reviewed by Bergström and Larsson [6] and Abramov et al. [7]. In summary, solubility can be predicted using either of two methods: Quantitative Structure Property Relationships (QSPR), which includes the GSE, and physics-based methods based on modelling of the thermodynamic cycle. The GSE, physics-based methods and a few QSPRs build on the three key steps taken when a molecule transitions from the crystalline state into an aqueous solution [6], i.e. a) dissociation from the crystal lattice [main descriptor: the melting point (MP)], b) preparation of a solvent cavity for incorporation of the molecule [main descriptor: the molecular weight (*M*_W_)] and c) insertion of the molecule in the solvent cavity and interactions with water [main descriptor: the logarithm of the partition coefficient between octanol and water (log *P*)]. In general, higher values for each of these three descriptors result in lower solubilities, but they are not by themselves providing perfect explanations of the individual steps in the solubilization process. For example, cavity formation could be better described by the molecular volume, which in turn is closely correlated to molecular weight. It was recently demonstrated that the solid-state contribution is the limiting factor in the accuracy and predictive power of models of solubility [8]. Therefore, if the solubility of a series of compounds is mainly controlled by their crystal packing, it is difficult to obtain an accurate prediction [3]. In such situations the application of a quantum mechanical (QM)-based thermodynamic cycle approach has been suggested [7]. However, if the solubility of a compound is mainly governed by lipophilicity, it is easier to predict its solubility with good accuracy.

Drug discovery for difficult-to-drug targets often results in ligands that are large, highly lipophilic and semi-flexible compounds, i.e. compounds residing in the “beyond rule of 5” (bRo5) chemical space [9]. Development of such compounds is associated with high pharmacokinetic risks, low solubility being one of them [10]. In addition to the aforementioned difficulties, additional issues in the prediction of solubility are encountered when dealing with bRo5 compounds. First, the low amount of experimental data available in the public domain limits the generation of models. Second, many drugs in this space display chameleon-like behaviour (i.e. they adapt their properties to the environment) which originates from their semi-flexibility and results in dynamic formation of intra-molecular hydrogen bonds (IMHBs) [11] and/or shielding of polar surface area [12, 13]. This introduces an additional level of complexity that should be taken into account in any modelling procedure.

To provide an update about the current status of solubility modelling in the bRo5 chemical space we first review the few bRo5 solubility models described in the literature. Then we report some computational strategies we applied to model the solubility of a dataset of ten bRo5 drugs and drug candidates, and to a second larger dataset of natural product inspired macrocycles. Lastly, we have summarized some key findings and attempted to set up preliminary guidelines for how to obtain reliable solubility models for drug discovery in the bRo5 chemical space.

## Recent developments

We recently investigated the solubility of a structurally diverse set of 11 drugs residing far into the bRo5 chemical space [12]. The selected drugs consisted of erythronolides and rifamycin antibacterial agents, as well as HIV-1 and HCV NS3/4A protease inhibitors. As determined by X-ray crystallography each drug populated >2 different conformations (RMSD >1.4 Å). Due to the difficulties in predicting the relevant conformational space for bRo5 drugs [14], these experimentally determined conformations were used to assess the impact of using 3D descriptors when modelling solubility. Solubility determined at pH 7.4, where seven of the drugs were ionized, was used in the solubility models, i.e. *S* (solubility at a given pH where the molecules can be fully or partially ionized) and not *S*_0_ (solubility of the neutral species) was used. We found that aqueous solubility was explained to some extent by the 2D descriptor of polarity, i.e. TPSA (r^2^ = 0.53), but that the correlation improved substantially when descriptors calculated from the 3D structures of the drugs [15] were used. The best model was based on the conformation of each drug that had the maximum molecular 3D PSA (Max M 3D-PSA, r^2^ = 0.83), while use of solvent accessible 3D PSA provided inferior models. The positive slopes of the correlations support, as expected, that the larger the PSA, the more soluble the drug. Notably, only a small difference in the quality of the regression model was obtained when the minimum molecular 3D-PSA was used instead of the Max M 3D-PSA. This finding, together with the observation that use of solvent accessible 3D PSA provided inferior models, suggests that the overall polarity of the molecule, originating from sampling of multiple aqueous conformations, is the most predictive for solubility. Solubility was also very well modelled by experimental lipophilicity (i.e. log *D*_7.4_) since the correlation between log *S* and log *D*_7.4_ had r^2^= 0.82. However, the relationship between solubility and calculated lipophilicity was not sought.

Very recently Avdeef and Kansy investigated to what extent the solubilities of small, Ro5-compliant molecules can be used to predict the intrinsic aqueous solubility of large molecules in the bRo5 chemical space [16]. Three solubility models published for Ro5 compliant molecules [4] were used to predict the solubility of a set of 31 bRo5 compounds, for which carefully curated log *S*_0_ values have been reported. The GSE and the Abraham Solvation Equation failed to predict the solubility of the larger compounds in bRo5 space, whereas the Random Forest Regression (RFR) method provided better results. The three methods differ in the applied algorithm, but also in the number of descriptors. Three were used in the GSE, five or six in the Abraham Solvation Equation and about 200 in RFR. Notably, 3D structural information was not used, but the authors suggest that the use of 3D descriptors (e.g. lipophilicity) could significantly improve predictions, since flexibility and conformational preferences can be expected to be more important for big than for small molecules.

Cyclic peptides are useful model systems for mapping solubility in the bRo5 chemical space, and also of major interest as leads on drug discovery projects. Qualitative structure-solubility relationships have been reported for cyclic peptides, but to the best of our ability we have not found any specific quantitative models. For instance, Lokey and co-workers reported that small variations in the side chains of synthetic analogues of the cyclic peptide natural product sanguinamide A had a large effect on their aqueous solubility [17]. Interestingly, in depth studies of three of the cyclic peptides revealed that the one that displayed conformational flexibility had chameleon-like behaviour resulting in high solubility and cell permeability, where two rigid analogues had low solubility but retained the high permeability. Another paper from the same group further exemplified the importance of conformational flexibility and chameleon-like behaviour for conveying high solubility and permeability of cyclic peptides from the phepropeptin and epiphepropeptin series [18]. Overall, these studies suggest that flexibility and conformational preferences should be included in the prediction of the solubility of cyclic peptides, but a more general approach on how to do this in practice still remains to be described.

## Solubility models for a small set of bRo5 drugs

**Figure 1.**
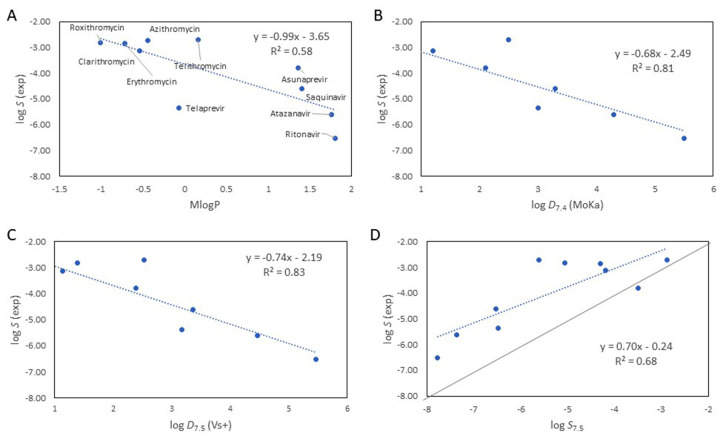
Solubility models for a dataset of 10 drugs in bRo5 space. Experimentally determined solubility at pH 7.4 (log *S*) and its correlation to **(a)** MlogP, implemented in the Ro5, **(b)** log *D*_7.4_ calculated in MoKa, **(c)** log *D*_7.5_ calculated using VolSurf+, and **(d)** log *S*_7.5_ calculated using VolSurf+.

We investigated additional methods to model solubility for 10 of the 11 drugs studied earlier by us (rifampicin was excluded because of its zwitterionic nature) [12]. As lipophilicity is one of the three major determinants of solubility according to the GSE, and as the size of these 10 drugs does not show a large variation (*M*_W_ 671-837 Da), we focused the modelling on log *P* and log *D* calculated by different methods (Figure 1, all data are in Table S1). MlogP, the 2D lipophilicity descriptor implemented in the Lipinski’s Ro5, provided a moderate correlation with log *S* (Figure 1A). As expected, significantly better correlations were obtained with log *D*_7.4_ calculated by MoKa (www.moldiscovery.com) and log *D*_7.5_ from VolSurf+ (VS+, www.moldiscovery.com), highlighting the importance of incorporating the charge of the drug in the models (Figures 1B and 1C). It is worth to note that inclusion of 3D structural information [log *D*_7.5_ (VS+)] did not significantly improve the statistical significance of the regression model found with 2D log *D* [log *D*_7.4_ (MoKa)] for this set of bRo5 drugs.

VolSurf+ also allows calculation of log *S*_0_ and log *S* at different pH values. We predicted log *S*_7.5_ (VS+) based on 3D descriptors derived from an average conformation produced by the software from the SMILES code of each of the 10 drugs. However, the correlation between log *S*_7.5_ (VS+) and the experimentally determined solubility (Figure 1D) was of lower quality than those obtained with log *D* descriptors (Figures 1B and 1C).

Besides VolSurf+ some other in silico tools apply 2D models to the prediction of solubility from SMILES codes. Most if not all of them have been set-up using datasets of small molecules having solubility values curated at different levels of quality. Nevertheless, considering the free availability and user-friendly interfaces we decided to evaluate them in the bRo5 chemical space. The SMILES codes of the 10 drugs were therefore submitted to admetSAR (http://lmmd.ecust.edu.cn/admetsar2/), ADMETLab (http://admet.scbdd.com/calcpre/index/), pKCSM (http://biosig.unimelb.edu.au/pkcsm) and Marvin Sketch (https://chemaxon.com/products/marvin). Notably, only log *S* calculated with Marvin Sketch provided a good linear relationship with the experimental data (r^2^=0.81 with log *S*, 0.59 with log *S*_0_; all the data are in Table S2). However, the slope and the intercept were significantly different from 1 and 0 (0.42 and -2.61, respectively) and thus the predicted values are not close to the experimental ones.

## A solubility classification model for a set of structurally complex macrocycles

**Figure 2.**
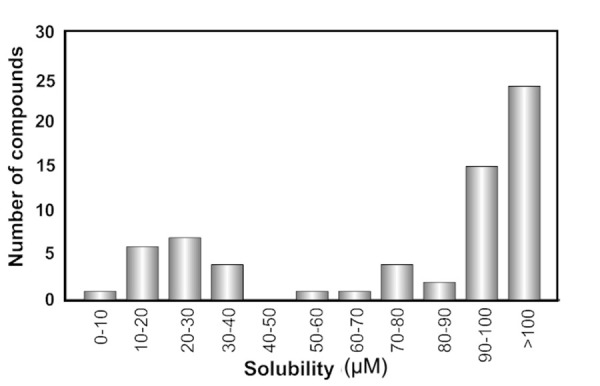
Distribution of the aqueous solubility for the de-novo designed macrocycles.

Previously, some of us determined the aqueous solubility, lipophilicity (log *D*) and permeability across Caco-2 cell monolayers for a set of 200 non-peptidic, de novo–designed macrocycles, the structures of which were inspired by natural products [19]. In-depth analysis of this dataset revealed that stereo- and regiochemistry can have a large influence on passive permeability and cellular efflux, whereas their impact on solubility appeared to be lower. Moreover, an appropriate conformational flexibility was concluded to be a highly desirable property that may provide compounds in bRo5 space with chameleonic properties that allow them to display both high aqueous solubility and high cell permeability. However, as structure-solubility relationships were not investigated for this set of macrocycles we now selected a subset consisting of 65 of the macrocycles for more detailed studies. The macrocycles in this set were selected by having no or very low efflux across Caco-2 cells (ER <2), i.e. by possessing one important property favourable for development of drugs in bRo5 space. The solubility distribution of the subset suggested that a classification rather than a regression strategy should be applied (Figure 2), and the threshold to distinguish soluble (47) from poorly soluble (18) compounds was fixed at 50 μM. This is a slightly more liberal cut-off than that proposed by GSK (30 μM) to distinguish compounds having poor from those having intermediate solubility [2].

In contrast to the 10 bRo5 drugs discussed above, descriptors for lipophilicity (log *P* or log *D*) failed to provide models for solubility for this set of de novo-designed macrocycles (data not shown) and therefore more complex methods were investigated. The CORINA (https://www.mn-am.com/online_demos/corina_demo) conformer of the charged and neutral forms of the selected macrocycles was obtained and conformational sampling was performed for both forms using OMEGA (https://www.eyesopen.com/omega). Then descriptors were calculated for (1) the 2D structure (which provided the 2D dataset), (2) the CORINA conformer (named 3D) and (3) the minimum energy conformer from OMEGA (named MEC). A pool of 2D descriptors were calculated for the first (2D) dataset, while both 2D and 3D descriptors were calculated for the 3D and MEC datasets. Random Forest (RF) classification models were built for data matrixes using WEKA v3.8 (https://www.cs.waikato.ac.nz/ml/weka/) and their quality was evaluated using the Matthews Correlation Coefficient (MCC), which takes into account true positives and negatives and returns a value between -1 and +1. A perfect prediction is characterized by a coefficient of +1, a random prediction by 0, while a completely incorrect prediction has an MCC of -1. In general, models having MCC values greater than 0.4 are considered to be predictive.

All models for this set of macrocycles were of good or high quality (Table 1, Leave-5-Out crossvalidation was used), but those obtained for the charged species, (MCC: 0.84 – 1.00) were superior to those for the neutral species (MCC = 0.43 – 0.92). This finding agrees well with the fact that most macrocycles in the dataset are predicted to be positively charged at pH 7.4. Notably, the best model (MCC = 1) was obtained for the charged forms using only 2D descriptors, while slightly inferior models were obtained when 3D descriptors were incorporated. The classification models were further assessed using an internal test set obtained by splitting the dataset into a training (50 macrocycles) and a test set (15 macrocycles). Again, the 2D-based RF model performed better than the models that included 3D information from conformational sampling (MCC = 0.71, Table S3). Eleven descriptors were found to have a high impact on the RF classification models, among which those describing lipophilicity, charge and surface area descriptors are the most important (Table S4).

## General considerations on solubility in the bRo5 chemical space

The previous sections clearly support that different bRo5 datasets can require different strategies for modelling of their solubility. For instance, the solubility of the 10 drugs in bRo5 space showed an excellent correlation with calculated log D only, whereas the solubility of the de-novo designed set of macrocycles required development of an advanced RF model. Overall, these observations highlight that models developed for specific, small datasets usually cannot be transferred to other datasets.

Another key observation is that the impact of ionization on solubility cannot be neglected when dealing with bRo5 compounds, just as for Ro5 compliant small molecules. Therefore, the p*K*_a_ of the investigated compounds should be accurately predicted before modelling solubility, which is a far from an easy and trivial task. Moreover, lipophilicity and polarity descriptors are needed to model solubility, but they should be specifically designed and validated for large and flexible compounds.

In principle, a 3D description of compounds in bRo5 space should be important for modelling solubility since conformational changes that expose surfaces with different properties could be required when a molecule dissociates from the crystal lattice and moves into solution. However, the examples discussed herein seem to suggest that the impact of the 3rd dimension on solubility is less important than for cell permeability. In fact, inclusion of 3D descriptors failed to improve the solubility models both for the 10 bRo5 drugs [12] and for the de-novo designed macrocycles [19]. In contrast, Avdeef and Kansy suggested that inclusion of 3D information might be important [16], and this is also observed for the cyclic peptides studied by Lokey and co-workers [17, 18]. Thus, it remains to be established if modelling of aqueous solubility is facilitated by methods for prediction of the conformations adopted in aqueous solution. However, we recently showed that reproducing experimental conformations by tools designed for conformational sampling of large and macrocyclic compounds is far from being an easy task [14].

Overall, more experimental data is needed to draw general conclusions about what the best approaches are for modelling the solubility of large and flexible compounds. This data is likely to be available in the pharmaceutical industry and partnering with academic researchers could be the preferred way to further analysis. We hope that this weakness can be overcome so that more reliable methods for prediction of the solubility of compounds lying in the bRo5 chemical space can be developed.



## Figures and Tables

**Table 1.** Summary of solubility classification models for the DOS macrocyclic dataset (Five-fold cross validation, #descriptors = number of descriptors of the model; TP = true positives, FN = false negatives; TN = true negatives; FP = false positives, MCC = Matthews Correlation Coefficient)

	Dataset	#descriptors	TP	FN	TN	FP	MCC
**Neutral**	2D	3	39	8	11	7	0.43
	3D	12	46	1	17	1	0.92
	MEC	3	46	1	16	2	0.88
**Charged**	2D	11	47	0	18	0	1.00
	3D	5	46	0	18	1	0.96
	MEC	10	46	1	15	3	0.84
