# Supplementary material for: Solubility prediction in the bRo5 chemical space: where are we right now?
Source: ADMET DMPK. 2020 Jul 8;8(3):207–14. doi: 10.5599/admet.834 (PMC8915608; doi:10.5599/admet.834)
Supplement: Supplementary file 1 [file admet-8-834-s001.pdf]

Supporting Information for

Solubility prediction in the bRo5 chemical space: where are we right now?

ADMET and DMPK (2020) doi: https://doi.org/10.5599/admet.834

Giuseppe Ermondi[1], Vasanthanathan Poongavanam[2], Maura Vallaro[1], Jan Kihlberg[2]* and Giulia Caron[1]*

[1] *Department of Molecular Biotechnology and Health Sciences, University of Torino, Quarello 15, 10135, Torino, Italy*

[2] *Department of Chemistry - BMC, Uppsala University, SE-75123, Uppsala, Sweden.*

Table S1. Lipophilicity data

Compound	exp log S	MlogP	log D (MoKa)	log D (Vs+)
Asunaprevir	-3.80	1.36	2.1	2.39
Atazanavir	-5.62	1.76	4.3	4.47
Azithromycin	-2.72	-0.44	-0.6	0.01
Clarithromycin	-3.13	-0.54	1.2	1.13
Erythromycin	-2.85	-0.72	0.4	0.57
Ritonavir	-6.52	1.8	5.5	5.46
Roxithromycin	-2.82	-1.01	0.7	1.39
Saquinavir	-4.61	1.4	3.3	3.37
Telaprevir	-5.37	-0.07	3	3.18
Telithromycin	-2.71	0.16	2.5	2.53

Table S2. Solubility calculated data

Compound	AdmetSAR2	ADMETLab	pKCSM	Marvin log S0	Marvin log S	log S (VS+)
Asunaprevir	-3.895	-4.157	-3.242	-7.46	-3.83	-3.49
Atazanavir	-2.903	-4.582	-3.849	-6.17	-6.17	-7.36
Azithromycin	-2.060	-3.201	-4.133	-1.62	0.38	-2.88
Clarithromycin	-2.322	-3.314	-4.387	-2.42	-0.81	-4.18
Erythromycin	-2.363	-3.045	-4.214	-2.27	-0.66	-4.30
Ritonavir	-3.225	-5.304	-4.194	-6.24	-6.24	-7.77
Roxithromycin	-3.028	-3.343	-3.819	-1.4	0.28	-5.07
Saquinavir	-3.225	-4.131	-3.936	-6.76	-5.65	-6.53
Telaprevir	-3.676	-3.699	-4.067	-7.4	-7.4	-6.49
Telithromycin	-3.494	-4.807	-2.987	-3.86	-2.61	-5.62

Table S3. Summary of solubility classification models for the DOS macrocyclic dataset (Test set, models of charged compounds)

Dataset	TP	FN	TN	FP	MCC
**2D**	10	0	3	2	0.71
**3D**	10	0	2	3	0.55
**MEC**	10	0	0	5	0

Abbreviations: TP: true positive, FN: false negative, TN: true negative, FP: false positive, MCC: Mathews correlation coefficient, MEC: Minimum energy conformer.

Table S4. List of descriptors contributed in the solubility models for DOS macrocyclic dataset

	Dataset	# variables	Descriptors
Neutral	2D	3	density, GCUT_PEOE_0, PEOE_VSA_FHYD
	3D	12	Vol, dens, VSA, CW3, CW5, HB3, ID8, R, W2, W6, Wp2, Wp3
	MEC	3	CASA+, vol, VSA
Charged	2D	11	a_IC, GCUT_PEOE_0, logP, FNEG, FPNEG, FPOL, Q_RPC-, PPOS, SlogP, VSA2, vdw_area
	3D	5	MLP, globe, HB3, IW5, Wp3
	MEC	10	PyPSA, MLP, DASA, FASA+, FCASA+, glob, vol, VSA, D1, HB2
